# Modulating presence and impulsiveness by external stimulation of the brain

**DOI:** 10.1186/1744-9081-4-33

**Published:** 2008-08-04

**Authors:** Gian Beeli, Gianclaudio Casutt, Thomas Baumgartner, Lutz Jäncke

**Affiliations:** 1 Institute of Psychology, Department of Neuropsychology, University of Zürich, Switzerland; 2 Institute for Empirical Research in Economics, Laboratory for Neuroeconomics and Social Neuroscience, University of Zürich, Switzerland

## Abstract

**Background:**

"The feeling of being there" is one possible way to describe the phenomenon of feeling present in a virtual environment and to act as if this environment is real. One brain area, which is hypothesized to be critically involved in modulating this feeling (also called presence) is the dorso-lateral prefrontal cortex (dlPFC), an area also associated with the control of impulsive behavior.

**Methods:**

In our experiment we applied transcranial direct current stimulation (tDCS) to the right dlPFC in order to modulate the experience of presence while watching a virtual roller coaster ride. During the ride we also registered electro-dermal activity. Subjects also performed a test measuring impulsiveness and answered a questionnaire about their presence feeling while they were exposed to the virtual roller coaster scenario.

**Results:**

Application of cathodal tDCS to the right dlPFC while subjects were exposed to a virtual roller coaster scenario modulates the electrodermal response to the virtual reality stimulus. In addition, measures reflecting impulsiveness were also modulated by application of cathodal tDCS to the right dlPFC.

**Conclusion:**

Modulating the activation with the right dlPFC results in substantial changes in responses of the vegetative nervous system and changed impulsiveness. The effects can be explained by theories discussing the top-down influence of the right dlPFC on the "impulsive system".

## Background

When we are watching a movie, reading a book or playing a computer game we sometimes experience these variants of virtual reality as if they were real. This subjective sensation of presence is referred as "the feeling of being there". From an earlier EEG (electroencephalography) study [1] we know that activations in certain brain areas (especially in the prefrontal cortex) are negatively correlated with the subjective feeling of presence in another space (spatial presence). The involvement in a virtual scene can be measured by questionnaires (e.g. MEC-SPQ [2]). Moreover, psychophysiological measures (e.g. electro-dermal activity or heart rate variations) are also used to indicate different presence states in a virtual environment (VE) of a person. Mostly, a higher involvement in the virtual reality scenario is accompanied by enhanced responses of the vegetative nervous system such as electrodermal responses and heart rate [1,3-5].

In this study we will use "transcranial direct current stimulation" to modulate brain activation during the confrontation with a virtual reality scenario. "Transcranial direct current stimulation" (tDCS) non-invasively modulates the excitability of a brain region of interest by altering neuronal membrane potentials [6,7]. Anodal tDCS has been found to increase cortical excitability and the potentiation of N-methyl-D-aspartate (NMDA) receptor efficacy, while cathodal tDCS has been found to decrease cortical excitability. Several studies have shown that the effects caused by tDCS last several minutes beyond the period of tDCS application [6-9]. Until now several studies have shown that tDCS can modulate cognitive and behavioral skills associated with the targeted brain area. For example, anodal tDCS to the left prefrontal cortex was found to increase working memory performance [10] and verbal fluency [11]. Anodal tDCS to the motor cortex contralateral to stroke patients' paretic arm facilitated temporary motor recovery [12]. In addition, anodal stimulation of the left motor cortex in healthy subjects improved right-hand performance [13]. A very recent study demonstrated that anodal stimulation to the supramarginal gyrus enhanced tone memory performance in musical novices [14].

In the context of these findings the question arises, whether the feeling of presence can be influenced by applying tDCS to brain areas known to be involved in the control of presence. In this study we focus on the dorso-lateral prefrontal cortex (dlPFC), which is known to be involved in controlling many higher-order behaviors. Typically it has been shown that this area is involved in selecting a possible range of responses and suppressing inappropriate ones [15]. In addition, it has been shown that this area is critically involved in the inhibition (and control) of impulsive behavior controlled by other brain-regions (e.g. the brainstem, basal ganglia; this system is sometimes called the „impulsive system") [16].

Thus, we anticipate that the dlPFC will be involved in the modulation of presence experience. If the dlPFC is activated there will be strong top-down control available inhibiting the automatically evoked presence feeling by the "impulsive system". When the dlPFC is deactivated the "impulsive system" can unfold its bottom-up activation with less top-down control of the dlPFC. If the dlPFC is indeed the critical area modulating presence feeling during the exposure of virtual environments the differential presence experience in kids, adolescents, and adults can be explained on the basis of the late maturing dlPFC [17]. The late myelination of the dlPFC can partly explain why adolescents' behavior is characterized by motivational difficulties, impulsivity and addiction (also in the context of video games and virtual scenes) [18].

In our study we modulated the right dlPFC with tDCS while participants were watching a virtual roller coaster scene. In order to further evaluate the success of this modulation, we also conducted a classical Go-Nogo task. The performance in this test depends on the functioning of dlPFC [19] and indicates the degree of impulsivity. There is evidence, that the task performance in the Go-Nogo task can be influenced by tDCS application to the left dlPFC [20] and with other methods also on the right dlPFC (transcranial magnetic stimulation (TMS) [21]). In addition, it has been shown that the right dlPFC is involved in controlling risk-taking behavior [22,23] and reciprocal fairness [24].

We hypothesize that the feeling of being present in the virtual environment is enhanced if the excitability (and thus the activation) of the dlPFC is decreased. In addition, lowered activation within in the dlPFC should also be accompanied by higher impulsiveness as measured with the Go-Nogo-task. On the other hand, if the excitability of the dlPFC (and thus the activation) is increased this should lead to a lowered presence experience and reduced impulsiveness.

## Methods

### Subjects

Thirty-five (17 female, 18 male) subjects participated in the experiment. Most of them being students of the University of Zurich. The mean age was 24.9 yr (standard deviation: ± 3.7 yr). All of the participants were classified as being consistent right-handed (CRH) using the Annett hand preference questionnaire [25]. No subject reported a history of neurological or psychiatric diseases and gave their informed consent for the participation in the experiment.

### tDCS application

In order to prevent an interaction between the two brain-hemispheres we decided to constrain tDCS to one hemisphere. In pilot experiments in our lab we found slightly stronger correlations between presence experience and brain activation on the right dlPFC than on the left side. Therefore, we only applied tDCS to the right dlPFC. The application side was at the FC3 electrode position of the international EEG 10–20-System. In order to constrain tDCS application to one hemisphere the reference electrode was placed on the ipsilateral mastoid. For tDCS application the "DC stimulator" by Eldith^© ^ was used. The constant current was applied using two saline-soaked electrodes with a surface of 35 cm^2^. During the anodal tDCS mode, the anode electrode was positioned on FC3 and the cathode electrode on the ipsilateral mastoid. During the cathodal condition, the two electrodes were switched (cathode over FC3, anode over ipsilateral mastoid). tDCS application lasted 5.5 min at a constant current intensity of 1.5 mA. The system automatically turned off the stimulation when the electrical resistance was too high. For sham stimulation the stimulator was switched off.

### Virtual roller coaster

**Figure 1:**
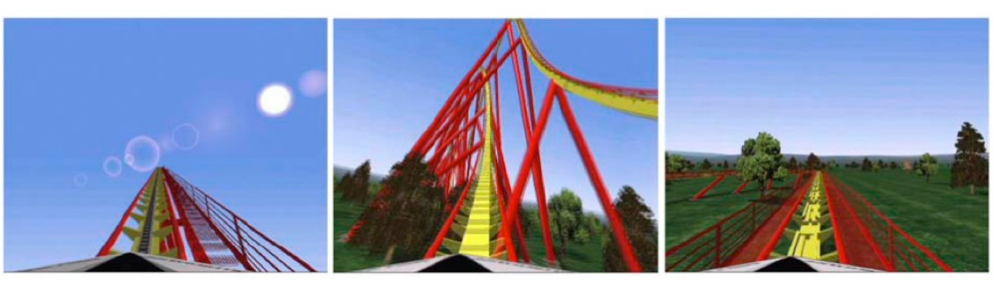
Example still figures of the used rollercoaster scenario. Ascending phase (left, 30 s), dynamic phase (middle, 60 s), end phase (right, 12 s).

The subjects were sitting on a chair while watching three different rollercoaster scenarios on a 22-inch computer screen placed at a distance of 60 cm in front of them. The rollercoaster scenarios were taken from a commercially available rollercoaster simulation software . Realistic driving noises were presented on loudspeakers. Every scenario consisted of three different phases. It started with an "ascending phase" (30 s) followed by a "dynamic phase" with movements in different dimensions and very high speed (60 s) and an "end-phase" with low speed and without inclination (Figure 1).

### Psychophysiological measures

During the roller coaster ride electro-dermal activity (EDA) and the electro-myogram (EMG) were registered. The EDA and EMG measurements were conducted using a commercially available device (PAR-PORT; Hogrefe Company, Germany). For EDA recording, electrodes were attached to the thenar and hypothenar areas on the palm of the left hand. EDA activity was quantified using two different measures. First, we measured skin conductance responses (SCR) to the roller coaster scenario. In addition, we measured skin conductance level (SCL) to measure the tonic level of skin conductance during the experimental sessions. SCL was measured as log-transformed mean EDA amplitude (log [EDAsumamp+1]). Log-transformation was used to normalize the SCL data. The EMG electrodes were attached at the left eyebrow muscle (musculus corrugator supercilii) and quantified as mean tonic EMG activity level at this site during the different experimental conditions.

### Go-Nogo task

The Go-Nogo task was taken from a German standard battery used to test several executive and attentional functions (Testbatterie zur Aufmerksamkeitsprüfung, TAP, [26]). This test consisted of 5 types of stimuli including lines in different directions. The subjects were required to press a button if one of the two defined target stimuli were presented. In total 100 stimuli were presented, 40 of them were target stimuli. The number of false alarms (FA, button-press when seeing a non-target stimulus) indicates the degree of impulsivity.

### Questionnaires

Since presence is a subjective feeling (first person) it is also necessary to use questionnaires asking the subjects for their particular presence feeling during the different conditions. We used an adapted version of the spatial presence questionnaire MEC-SPQ [5]. The questionnaire was presented to the subjects immediately after the rollercoaster ride. Participants indicated their degree of presence on a visual analog scale. Moreover, the SAM (Self Assessment Manikin) was administered after each roller coaster ride in order to control for mood changes during the tDCS application. With the SAM experienced arousal and valence during the roller coaster presentation was measured. Although not being the main focus of this paper we used some subscales ("thrill and adventure seeking scales"; TAS) of the „sensation seeking questionnaire" to control whether this trait might have an influence on presence experience [27,28].

### Design

**Figure 2:**
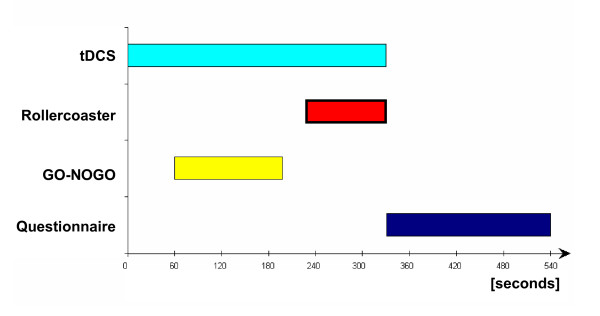
Experimental design. Sequence of the different tasks and tDCS applications. The time scale is in seconds. This sequence was repeated three times per subject for the three stimulating conditions (sham, anodal, cathodal).

We used a repeated measurements design in which every subject was randomly assigned to the three different conditions (anodal, cathodal, sham). During each condition the subjects were exposed to the roller coaster scenario after they have received different tDCS treatments. Each condition comprised the tDCS application, followed by the Go-Nogo task, the roller coaster presentation and the final questionnaire measurement (Figure 2). Between each condition there was a break of 3.5 minutes without any task and tDCS application.

### Statistical analysis

The number of false alarms, SCR, SCL, as well as ratings of valence, arousal, and presence were subjected to one-way repeated measurements ANOVAs with three levels (sham, anodal, and cathodal). Before ANOVA analysis the variances were evaluated for homoscedasticity and we also checked the data for normal distribution. There was no significant deviation from homoscedasticity making it unnecessary to use specific corrections (e.g., Greenhouse Geisser corrections). In addition, the data were also evaluated whether they deviate from normal distribution. Since there were no strong deviations from normal distribution we deemed the ANOVA as an appropriate method to analyze this data set. In case of significant main effects subsequent post-hoc t-tests were conducted using the Bonferroni-Holm procedure [29]. A p value < = 0.05 was used as statistical threshold.

## Results

### Go-Nogo task

**Figure 3:**
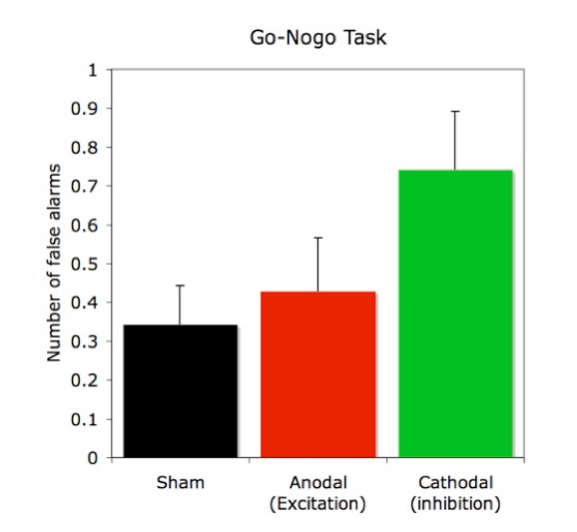
Number of false alarms (FA) in the different conditions in the Go-Nogo task. Applying cathodal tDCS to the right dlPFC led to an enhanced number of FA (p < .03) compared to sham and anodal-Stimulation. Depicted are means of FA (± SE).

Figure 3 shows the results of the Go-Nogo task separately for the three experimental conditions. During cathodal tDCS participants generated more often false alarms indicating a tendency for impulsive behavior. There was no change in performance during anodal stimulation. Subjecting the number of false alarms to a one-way repeated measurements ANOVA revealed a significant between-condition difference for the number of false alarms (F(2,68) = 3.653; p = 0.03). Subsequently conducted post hoc tests revealed significant differences between false alarms obtained during "sham" vs. "cathodal" (p = 0.032) and "anodal"- vs. "cathodal" (p = 0.033).

### Psychophysiological measures

**Figure 4:**
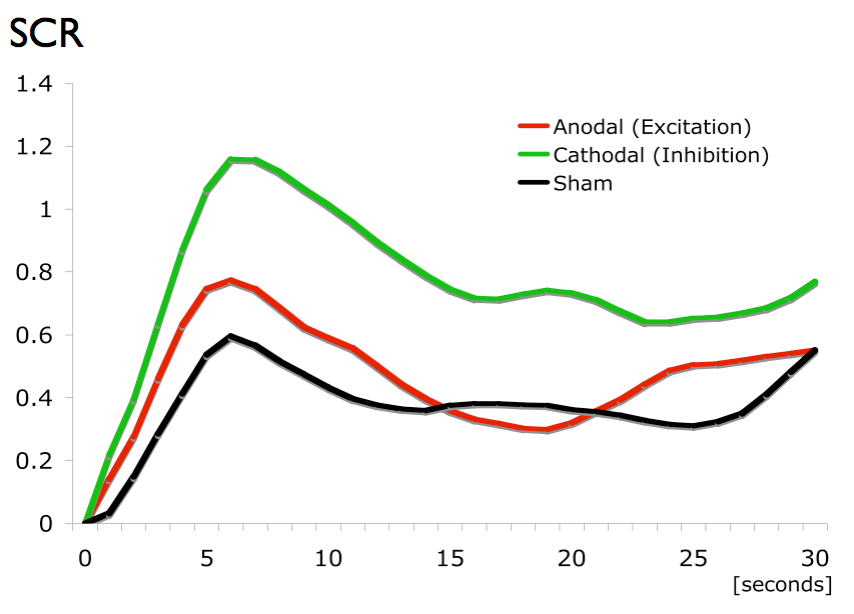
Skin conductance level of the first 30 seconds of the rollercoaster ride. Cathodal tDCS application (inhibition) to the right dlPFC led to an enhanced skin conductance response (SCR).

Due to artifact contamination only data of 29 participants could be used for analysis of psychophysiological measures. The EMG measure during the roller coaster ride showed no significant difference during the three tDCS conditions. For SCR a significant between-condition difference emerged. Figure 4 shows a clear SCR at the start of the virtual roller coaster ride. In the first 30 seconds of the rollercoaster ride (ascending phase), subjects showed stronger SCR during cathodal tDCS (F(2,56) = 3.237; p = 0.047; cathodal > sham: p = 0.021). The one-way ANOVA conducted for the SCL data did not reveal significant differences (F(2,56) = 3.016; p = 0.057).

**Figure 5:**
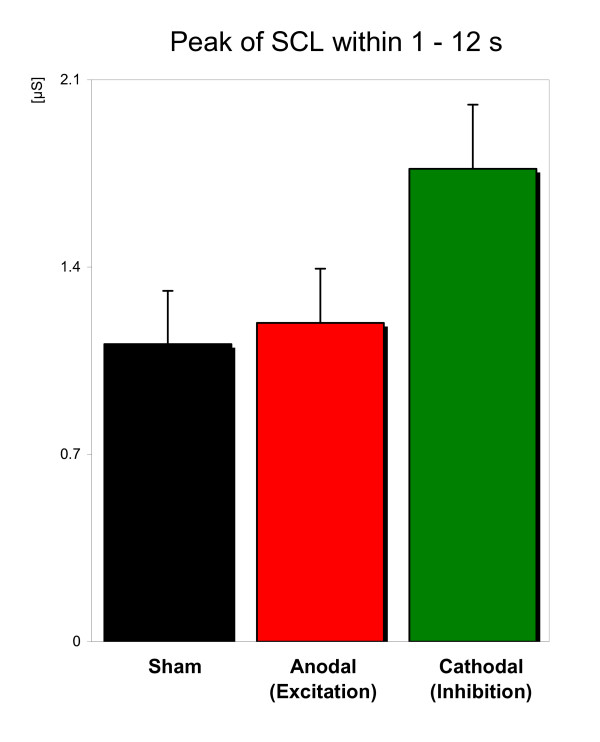
Peak of skin conductance level (maximum SCL) in the first 12 seconds of the roller coaster ride. Cathodal tDCS application (inhibition of the dlPFC) leads to significantly enhanced maximum SCL (p < .01) during the ascending phase in the virtual roller coaster compared to sham and anodal stimulation. Depicted are means of the SCL (± SE).

Figure 5 shows the mean peak SCL measured during the first 12 seconds of the roller coaster ride. Peak SCLs were significantly different in the three conditions (F(2,56) = 4.958 p = 0.01) with a higher peak during cathodal stimulation vs. anodal stimulation (p = 0.005) and vs. sham stimulation (p = 0.012).

### Presence- and personality questionnaires

The questionnaire data showed no significant differences between the different tDCS conditions. Interestingly, there was no significant correlation between the subscale "thrill and adventure seeking" (TAS) and the SCR measures (p > .33). The self-assessment-manikin (SAM) showed no differences with respect to the experienced valence of roller coaster scenarios during the different conditions (F(2,66) = 1.617; p = 0.206). However, there was a tendency for slightly increased subjective arousal levels during tDCS application compared to sham stimulation (F(2,66) = 2.532; p = 0.087).

### Correlation between Go-Nogo task performance and SCR

There was also a significant correlation between the number of false alarms (taken as a measure for impulsiveness) and the SCR measures (r = 0.42, p < 0.02). Thus, if participants act more impulsively in the Go-Nogo task, they also show stronger SCR measures in the ascending phase of the roller coaster ride.

## Discussion

Our study demonstrates that the application of cathodal tDCS to the right dlPFC modulates the degree of impulsivity (as measured with the number of false alarms in the Go-Nogo task). It follows from the current interpretation of the effect of cathodal tDCS on the neural system underlying the cathode that cathodal tDCS downregulates the dlPFC, with a resultant reduction in neural activation in this area. In line with this interpretation, we suggest that the dlPFC exerts less top-down control over the "impulsive system", increasing the likelihood therefore of impulsive behavior [for a summary see [30]]. Applying anodal tDCS to the dlPFC did not affect impulsiveness as indicated by Go-Nogo performance. We hypothesized at the beginning of the study that this kind of tDCS application would lead to increased neural activation of the dlPFC, this in turn would facilitate increased top-down regulation of the "impulsive system" in the form of reduced impulsiveness. The reason that we did not obtain this result is probably due to the fact that the task was too easy with too few false alarms, even in the sham condition. Thus, there was a kind of "floor effect" without any opportunity to decrease the number of false alarms. This might also explain the different findings in previous studies using a more difficult and slightly different versions of the Go-Nogo task [20,21].

Besides the differential effect of tDCS on the number of false alarms, we also obtained different results for the skin conductance responses (SCR) used to indicate the reactivity of the vegetative nervous system. Application of cathodal tDCS to the dlPFC elicited increased SCRs while the subjects were exposed to the roller coaster scenario. This differential SCR was only present in the first phase of the roller coaster ride during which the virtual cab was ascending to the top of the roller coaster course (ascending phase). The strong skin conductance response during the ascent phase of the roller coaster ride might be associated with the anticipation of the following dynamic phase with its ups and downs and with the experience or expectation of bodily arousal in a real roller coaster.

The correlation between the number of false alarms in the Go-Nogo task and the SCR measures indicates that impulsive behavior and autonomic responses can be influenced by tDCS application, and that both reactions might depend on the activation in the right dlPFC. However, further investigation is needed to develop a better understanding of the relationship between the inhibition of impulsive behavior and vegetative reactions.

The fact that the personality trait TAS (thrill and adventure seeking) had no impact on our measures (e.g., skin conductance or number of false alarms) indicates that the application of tDCS is independent of the "sensation seeking" personality. Nevertheless, there might still be different effects on patients as found in patients with major depression [20].

A further important result of the present study is that there are significant differences in vegetative reactions in the hypothesized direction associated with tDCS application, but that the subjective reports (measures with questionnaires) did not differ for the different conditions. This shows that subjective measures might not be reliable in the context of presence research (especially because the involvement in a VE requires low cognitive control) and that brain stimulation can lead to a change in bodily reactions without influencing subjective reports.

## Conclusion

Application of tDCS to the right dlPFC can influence the vegetative reactions while watching a virtual roller coaster scene as well as the number of false alarms in a standard Go-Nogo discrimination task commonly used as a behavioral measure of impulsivity. The measured vegetative effects during viewing of the virtual roller coaster ride and concomitant tDCS application had no impact on self-reported experience of presence. The cathodal (inhibiting) condition leads to enhanced impulsivity and higher skin conductance responses. There was no effect on skin conductance and impulsivity during the anodal (exciting) condition.

## Competing interests

The authors declare that they have no competing interests.

## Authors' contributions

GB participated in the design of the study, performed the statistical analysis and drafted the manuscript. GC participated in the design, carried out the experiments and performed the statistical analysis. TB participated in the design. LJ participated in the design, the statistical analysis and drafted the manuscript. All authors read and approved the final manuscript.
